# A Case of Familial Mediterranean Fever With Severe Attacks During Withdrawal Bleeding After Controlled Ovarian Hyperstimulation

**DOI:** 10.7759/cureus.76168

**Published:** 2024-12-21

**Authors:** Mio Tatara, Yuji Tanaka, Akie Takebayashi, Shunichiro Tsuji, Takashi Murakami

**Affiliations:** 1 Department of Obstetrics and Gynecology, Shiga University of Medical Science, Otsu, JPN; 2 Department of Obstetrics and Gynecology, Shiga University of Medical Science, Ostu, JPN

**Keywords:** assisted reproductive technology, colchicine, controlled ovarian hyperstimulation, familial mediterranean fever, in vitro fertilization, menstruation-associated attack, withdrawal bleeding

## Abstract

Familial Mediterranean fever (FMF) is an autoinflammatory disease characterized by periodic fever, serositis, and arthritis. In women, FMF attacks can sometimes be triggered by the menstrual cycle. Once diagnosed, prophylactic treatment with colchicine is generally recommended. Here, we report the case of a 34-year-old nulligravid Japanese woman who met the Tel-Hashomer criteria for FMF with menstruation-associated attacks and experienced a severe FMF episode following controlled ovarian hyperstimulation (COH) for in vitro fertilization (IVF) without prophylactic colchicine. We also discuss management strategies for menstruation-associated FMF in patients who do not receive prophylactic therapy. Although prophylactic colchicine treatment was advised, the patient declined, and a plan for symptomatic management was established. She was referred to our clinic for infertility and subsequently underwent IVF due to endometriosis. At the initial examination, an increase in follicle-stimulating hormone (FSH) levels was observed, and endogenous endocrine function was regulated by administering estrogen and progestin. She experienced two withdrawal bleeding episodes without FMF attacks.

Subsequently, COH using a gonadotropin-releasing hormone (GnRH) antagonist protocol was initiated, and oocyte retrieval was performed when her estradiol (E2) level reached 953 pg/mL. Immediately after the withdrawal bleeding following oocyte retrieval, she experienced her most severe FMF attack to date, presenting with fever above 38°C, diffuse abdominal pain, vomiting, and joint pain. To prevent further FMF attacks during the waiting period before embryo transfer, colchicine or dienogest was administered, effectively suppressing additional episodes. This case suggests that in patients with menstruation-associated FMF, withdrawal bleeding after COH may strongly provoke FMF attacks due to the abrupt decline in estrogen levels. It underscores the importance of prophylactic therapy with colchicine or other agents during assisted reproductive technology (ART). Furthermore, when prophylactic treatment is not feasible, alternative strategies such as fresh embryo transfer should be considered.

## Introduction

Familial Mediterranean fever (FMF) is an autoinflammatory disease characterized by periodic fever, serositis, arthritis, and various other symptoms. Like tumor necrosis factor receptor-associated periodic syndrome (TRAPS), cryopyrin-associated periodic syndrome (CAPS), and hyperimmunoglobulin D syndrome (HIDS), it is classified as a hereditary periodic fever syndrome within the category of autoinflammatory diseases. The Tel-Hashomer criteria are widely used as a standard diagnostic tool for FMF [1]. Since recurrent, untreated attacks can lead to amyloidosis, it is crucial to initiate preventive treatment promptly once the diagnosis is confirmed [1]. Colchicine is effective in preventing FMF attacks in more than 90% of cases and can be safely administered to women who wish to conceive, as it carries a low risk of teratogenicity [2]. For colchicine-resistant cases, agents such as canakinumab (which targets interleukin-1 [IL-1]) and tumor necrosis factor-α (TNF-α) inhibitors (e.g., infliximab, etanercept) have shown efficacy.

FMF can be triggered by various factors, including infections, trauma, and stress [3]. Studies have identified that some patients experience FMF episodes associated with their menstrual cycles. Compared to those without menstrual-associated attacks, patients with menstruation-associated FMF attacks tend to be younger at onset and diagnosis, have a higher incidence of peritonitis, and show a higher prevalence of endometriosis [4]. Although the underlying mechanisms are not fully understood, fluctuations in estrogen levels are hypothesized to play a role in these menstrual cycle-associated attacks.

Although FMF is most commonly seen in populations from the Mediterranean region, patients have also been identified in East Asia, including Japan [5-9]. The total number of Japanese FMF patients was estimated to be 292 (95% confidence interval: 187-398) based on the primary nationwide survey [5].

In this report, we present a case of a female patient diagnosed with FMF featuring attacks associated with her menstrual cycle. Despite medical advice emphasizing the low teratogenic risk of colchicine, the patient refused prophylactic treatment due to concerns about teratogenicity. She subsequently experienced a severe FMF attack during withdrawal bleeding following ovulation induction.

## Case presentation

A 34-year-old nulligravid Japanese woman with a history of hypothyroidism, managed with levothyroxine (50 µg/day), presented with a six-month history of recurrent fevers exceeding 38°C, joint pain, and non-localized abdominal pain coinciding with her menstrual cycle, which was regular with a 28-day interval. These symptoms prompted her to seek medical attention. During febrile episodes, marked increases in C-reactive protein (CRP) and serum amyloid A (SAA) levels were observed, which decreased once the fever subsided (Table 1). Her fevers typically have resolved within two days. The patient met the Tel-Hashomer criteria (Table 2) and was diagnosed with FMF. At her request, tests for Mediterranean fever (MEFV) gene mutations (M694I, M694V, V726A, E84K, E148Q, L110P, P369S, R408Q, R202Q, G304R, S503C) were performed, all of which were negative.

**Table 1:** Blood test results CRP - C-reactive protein; SAA - serum amyloid A; anti-SS-A/Ro -  anti-Sjögren's syndrome-related antigen A; Anti-SS-B/La - anti-Sjögren's syndrome-related antigen B (La); IgG - immunoglobulin G; C3 - complement component 3; C4 - complement component 4

Timing of examination	Reference values	At attack	At non-attack	Examination of autoinflammatory disease
CRP (mg/dL)	0.00-0.14	6.61	0.02	
SAA (mg/dL)	0.0-3.0	666	4.1	
Anti-nuclear antibody (ANA)				negative
Anti-DNA antibody				negative
Anti-SS-A/Ro antibody				negative
Anti-SS-B/La antibody				negative
Ferritin (ng/mL)	3.6-114			30.2
C3 (mg/dL)	73-138			113
C4 (mg/dL)	11-31			32
IgG (mg/dL)	861-1747			1701

**Table 2:** Tel-Hashomer criteria for the diagnosis of familial Mediterranean fever A definite diagnosis is established if the patient meets two or more major symptoms or one major plus two minor symptoms. FMF - familial Mediterranean fever Source: [1]

Major criteria	Minor criteria
Recurrent febrile episodes with serositis (peritonitis, synovitis or pleuritis)	Recurrent febrile episodes
Amyloidosis of AA type without a predisposing disease	Erysipelas-like erythema
Favorable response to regular colchicine treatment	FMF in a first-degree relative

Although prophylactic treatment with colchicine, which has a low risk of teratogenicity, was proposed, the patient refused due to fears of teratogenic effects. Consequently, colchicine was not administered.

No other cause for infertility was identified apart from endometriosis. Her anti-Müllerian hormone (AMH) was 1.51 ng/mL. The patient had previously undergone more than six cycles of timed intercourse therapy without achieving pregnancy and was referred to our reproductive medicine division for infertility treatment. Magnetic resonance imaging (MRI) revealed multiple endometriotic cysts in the left ovary, the largest measuring 22×16×15 mm, as well as small endometriotic cysts in the right ovary. In vitro fertilization (IVF) was planned to address her endometriosis.

At the initial consultation, elevated follicle-stimulating hormone (FSH) levels (FSH=21.8), prompting the administration of estrogen and progestin for two cycles to stabilize her endogenous endocrine function. She experienced two withdrawal bleeding episodes during this period, with no FMF attacks occurring. After normalization of her FSH levels, controlled ovarian hyperstimulation (COH) using a gonadotropin-releasing hormone (GnRH) antagonist protocol was initiated. A total of 3900 IU of gonadotropin was administered, and GnRH antagonist treatment began on the eighth day of COH. When her estradiol (E2) level reached 953.8 pg/mL, 250 µg of choriogonadotropin alfa was administered. Thirty-five hours later, four oocytes were retrieved under transvaginal ultrasound guidance. The ovarian endometriotic cyst was not punctured during the oocyte retrieval.

Eleven days after oocyte retrieval, the patient experienced withdrawal bleeding followed by a fever exceeding 38°C, non-localized abdominal pain, vomiting, and general joint pain, constituting her most severe FMF attack to date. Her CRP level had risen to 10.7 mg/dL. Transvaginal ultrasound revealed a normal-sized uterus and at least two known endometriotic cysts in the left ovary. No developing follicles larger than one centimeter or ascites were detected, and there was no adnexal tenderness. Antibiotics were administered as an infection could not be initially ruled out. Subsequently, blood, urine, and vaginal secretion cultures were all negative.

**Figure 1:**
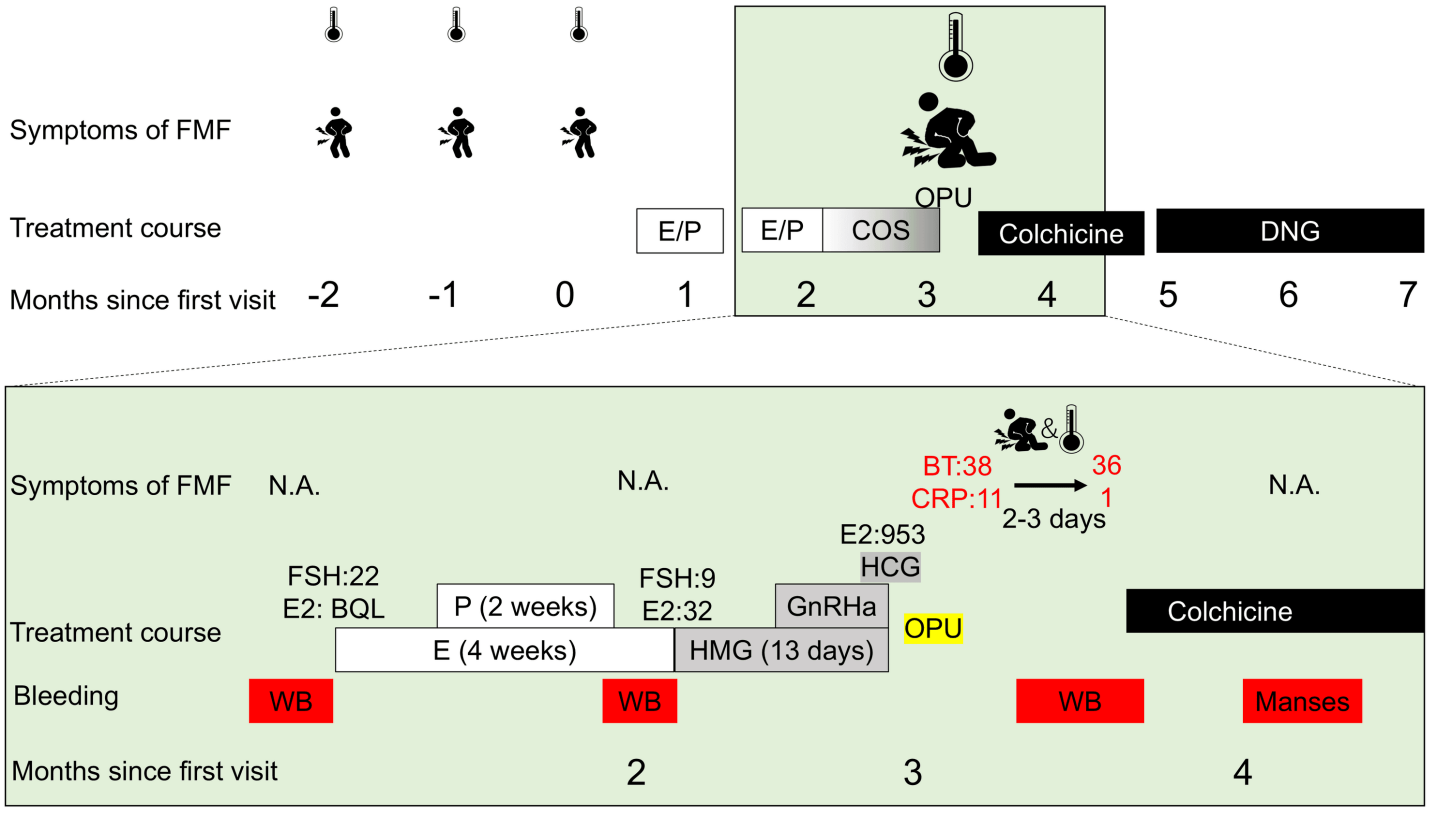
The clinical course of the case E2 - estrogen; P - progestin; OPU - ovum pick-up; DNG - dienogest; N.A. - not applicable; BT - body temperature; HMG - human menopausal gonadotrophin; WB - withdrawal bleeding; BQL - below quantification limit; FSH - follicle-stimulating hormone; FMF - familial Mediterranean fever; CRP - C-reactive protein; GnRHa - gonadotropin-releasing hormone agonist; COS - controlled ovarian stimulation

The patient's fever resolved within two days, and both her symptoms and inflammatory markers improved rapidly. She was scheduled for sinus surgery and hysteroscopic polypectomy prior to embryo transfer. To prevent FMF attacks during the waiting period, colchicine (0.5 mg/day) was initiated. No FMF attacks occurred following the return of spontaneous menstruation; however, she discontinued colchicine due to diarrhea and switched to dienogest therapy, during which no attacks were observed. Figure 1 illustrates the clinical course of this case.

## Discussion

We present a case of a woman with menstruation-associated familial Mediterranean fever who underwent IVF without prophylactic colchicine treatment. After receiving estrogen and progestin, she experienced withdrawal bleeding without any FMF attacks. However, following oocyte retrieval, the subsequent withdrawal bleeding triggered a severe FMF attack, which resolved with the administration of dienogest (DNG). This finding suggests that in menstruation-associated FMF, withdrawal bleeding after COH may strongly precipitate FMF attacks due to a rapid decrease in estrogen levels.

It has been reported that, in FMF patients with menstruation-associated attacks, episodes are suppressed when estrogen levels remain stable. For example, in patients receiving low-dose estrogen-progestin (LEP), attacks are prevented even when withdrawal bleeding occurs [10]. Similarly, during pregnancy [11] or DNG treatment [4], attacks can be controlled without prophylaxis. In our case, no attack occurred after withdrawal bleeding induced by estrogen and progestin prior to COH, and no attack occurred during DNG therapy, likely due to relatively low and stable estrogen levels. By contrast, after COH, withdrawal bleeding led to a severe flare, possibly triggered by the rapid decline in estrogen following oocyte retrieval.

Although previous reports have described IVF in menstruation-associated FMF patients, these patients typically received colchicine prophylaxis before initiating assisted reproductive technology (ART), likely preventing attacks [12-14]. Our findings indicate that withdrawal bleeding after COH can serve as a potent trigger for FMF flares in menstruation-associated FMF patients not receiving prophylactic treatment. This insight is clinically significant for patients seeking pregnancy via IVF. Given reports suggesting that FMF patients may have a lower ovarian reserve and reduced pregnancy rates compared to the general population, this observation becomes even more critical [15].

In some menstruation-associated FMF patients, attacks are suppressed by LEP or DNG until pregnancy is desired [4,10]. For such patients, when undergoing COH, administering colchicine as a preventive measure after discontinuing hormone therapy is advisable. In cases like ours, where the patient is not on prophylactic treatment due to side effects or personal preference and is managed symptomatically, it is worth noting that pregnancy itself may suppress FMF flares, and a fresh embryo transfer could be considered. In any scenario, since ART involves substantial fluctuations in estrogen levels, preventive interventions are necessary for FMF patients undergoing these treatments.

The diagnosis of familial Mediterranean fever relies on a combination of clinical criteria and genetic testing. While genetic analysis can support the diagnosis by identifying mutations in the MEFV gene, the absence of mutations does not exclude FMF, as seen in this case. Although no FMF-associated genetic mutations were detected in this patient, the diagnosis was based on the internationally recognized Tel-Hashomer criteria. As part of the differential diagnosis, autoimmune conditions such as systemic lupus erythematosus (SLE) and Sjögren's syndrome were considered. Tests for antinuclear antibodies (ANA), anti-DNA antibodies, anti-Sjögren's syndrome-related antigen A (Ro) (anti-SS-A/Ro)antibodies, anti-Sjögren's syndrome-related antigen B (La) (anti-SS-B/La) antibodies, ferritin, complement component 3​​​​​​​ (C3), complement component 4 (C4), and immunoglobulin G (IgG) were negative or within normal ranges. Although not performed in this case, human leukocyte antigen​​​​​​​ (HLA) testing might also be considered in the differential diagnosis of FMF, as Behçet's disease, which occasionally presents with similar symptoms, should also be included. The gene responsible for FMF is MEFV, which encodes pyrin. While FMF is primarily inherited in an autosomal recessive manner, there are reports of autosomal dominant inheritance as well. The prevalence of specific MEFV gene mutations varies regionally; in the Mediterranean region, common mutations include M694V, M694I, V726A, and M680I, all of which are Exon 10 mutations [5]. In Japan, the genetic background differs from that of the Mediterranean region. Notably, the most common Mediterranean mutation, M694V, has not been reported in Japan [5]. Genetic abnormalities in Exon 2 and Exon 3 are said to be common in Japanese people [8]. Furthermore, approximately 15% of Japanese patients, including this case, do not exhibit any known genetic mutations [5].

A limitation of this report is the inability to completely exclude infection as the cause of fever during withdrawal bleeding after oocyte retrieval. However, based on the clinical and laboratory findings, we concluded that this episode was more likely an FMF attack rather than an infection. Laboratory results revealed negative cultures, while clinical symptoms included generalized abdominal pain extending to the upper abdomen and generalized joint pain, both consistent with episodes of serositis and arthritis as defined by the Tel-Hashomer criteria's major criteria. Notably, the patient lacked adnexal tenderness, a hallmark of pelvic inflammatory disease. Furthermore, as endometriotic cysts were not punctured, the risk of infection was considered low, which may further support the conclusion that this was an FMF attack.

## Conclusions

In menstruation-associated FMF patients not receiving prophylactic treatment, withdrawal bleeding after COH can be a strong trigger for FMF attack. For patients whose attacks are suppressed with hormonal therapy until conception is desired, or for those managed symptomatically due to concerns such as side effects or personal preference, careful consideration should be given to preventive measures. Fluctuations in estrogen levels during withdrawal bleeding may provoke severe attacks, making prophylactic treatment such as colchicine highly recommended. If prophylaxis is not feasible, fresh embryo transfer could be considered to reduce the risk of FMF flare-ups and mitigate the impact of estrogen-induced hyperestrogenism during COH.
